# Immune Profile Predicts Survival and Reflects Senescence in a Small, Long-Lived Mammal, the Greater Sac-Winged Bat (*Saccopteryx bilineata*)

**DOI:** 10.1371/journal.pone.0108268

**Published:** 2014-09-25

**Authors:** Karin Schneeberger, Alexandre Courtiol, Gábor Á. Czirják, Christian C. Voigt

**Affiliations:** 1 Leibniz Institute for Zoo and Wildlife Research, Berlin, Germany; 2 Freie Universität Berlin, Department Animal Behaviour, Berlin, Germany; CSIRO, Australia

## Abstract

The immune system imposes costs that may have to be traded against investment of resources in other costly life-history traits. Yet, it is unknown if a trade-off between immunity and longevity occurs in free-ranging mammals. Here, we tested if age and survival, two aspects associated with longevity, are linked to immune parameters in an 8 g bat species. Using a combination of cross-sectional and longitudinal data, we assessed whether total white blood cell (WBC) counts, bacterial killing ability of the plasma (BKA) and immunoglobulin G (IgG) concentration change with age. Furthermore, we asked if these immune parameters impose costs resulting in decreased survival probabilities. We found that WBC counts decreased with age both within and among individuals. IgG concentrations were higher in older individuals, but did not change with age within individuals. Furthermore, individuals with above average WBC counts or IgG concentration had lower probabilities to survive the next six months. High WBC counts and IgG concentrations may reflect infections with parasites and pathogens, however, individuals that were infected with trypanosomes or nematodes showed neither higher WBC counts or IgG concentrations, nor was infection connected with survival rates. BKA was higher in infected compared with uninfected bats, but not related to age or survival. In conclusion, cellular (WBC) and humoral (IgG) parts of the immune system were both connected to age and survival, but not to parasite infections, which supports the hypothesis that energetically costly immunological defences are traded against other costly life-history traits, leading to a reduced lifespan in this free-ranging mammal.

## Introduction

By exploiting host resources, parasites and pathogens inflict damages and therefore impose a negative effect on host fitness (e.g. [1]). Mounting and maintaining immune functions provide an animal with a set of cellular and biochemical defence mechanisms against potentially harmful agents, but such traits are also energetically costly (reviewed in [2]). Previous studies on potential trade-offs between immunity and other life-history traits focussed mainly on the relationship between immunity and reproduction. For example, it has been shown that the immune system may get impaired when energy has to be allocated largely to current reproductive events [3], [4]. In contrast, the relationship between immune response and other life-history traits such as longevity is largely unknown, particularly in free-ranging mammals.

Similarly to other eco-immunological questions, most evidence for the potential trade-off between immunity, survival and longevity have been studied in birds [5]–[7] and in laboratory mammals such as domestic mice [8]. In captive voles (*Microtus arvalis*) for example, it has been shown that immune challenges do not influence longevity, neither in males nor females [9], but such data is lacking on free-ranging mammals. Studies performed under laboratory conditions may result in reduced mortality (e.g. *ad libitum* food, constant veterinarian surveillance) and thus prolonged lifespans. Therefore, such studies may be biased regarding the strength of potential age-related trade-offs between immunity and longevity. For example, the relevance of a trade-off expressed late in life could be null for laboratory animals outliving their conspecific in the wild. To shed light on the functional relationship between the immune system and survival in mammals, studies on free-ranging animals are therefore urgently needed [10]. So far, studies on the link between selected immune components and survival in a free-ranging mammal have concentrated on a population of Soay sheep (*Ovis aries*). For example, it has been shown that antinuclear antibody concentrations were associated with reduced reproduction but increased survival during harsh winters [11]. Yet, how other aspects of the immune system are associated with longevity in mammals remains to be determined.

Besides survival, senescence is another important component characterising longevity in animals. With increasing age, irreversible physiological and molecular changes accumulate and impair the performance of individuals, including the immune system [12]. Evidence for a decrease in immune functions with age (immunosenescence) has been found in humans, domestic and species living in laboratory conditions [13]–[19]. Studies on senescence in free-ranging mammals are rare, probably due to difficulties in estimating an individual’s age in the field. To our knowledge, a cross-sectional study on immunosenescence in Soay sheep [20] is the only one comparing immune parameters of juvenile, adult and geriatric individuals in a free-ranging mammalian population. However, patterns from cross-sectional studies may be confounded by the selective disappearance of particular phenotypes with age and therefore, longitudinal data would be needed in order to shed light on trade-offs between branches of the immune system and aspects of longevity, such as survival and senescence [21], [22].

Here, we aim at testing potential trade-offs between immune components and survival, and furthermore ask if both cellular and humoral immune components change with age (immunosenescence) using a combination of cross-sectional and longitudinal data in a free-ranging population of male and female greater sac-winged bats (*Saccopteryx bilineata*). We make use of long-term data collected on this population, including data on longevity. We predict that aspects of the immune system decrease with increasing age in a longitudinal approach due to immunosenescence, and in a cross-sectional approach due to selective disappearance of individuals with overly high levels of immune components. Furthermore, we predict reduced survival in individuals that invest more than average in costly cellular aspects [23] of the immune system.

## Materials and Methods

### Ethics statement

Capturing and sampling of bats was approved by the Committee for Ethics and Animal Welfare of the Leibniz Institute for Zoo and Wildlife Research (Approval No. 2010-09-01), the scientific committee of “La Selva” Biological Station and by the national authorities of Costa Rica (No. 137-2010-SINAC and 168-2011-SINAC) and complied with the current laws of the country. *Saccopteryx bilineata* is listed as “least concerned” by the IUCN and not protected in Costa Rica.

### Study system

Greater sac-winged bats (*Saccopteryx bilineata*) show high roost fidelity and are therefore an ideal study organism for research questions related to ageing. At our Costa Rican study site, this species has been monitored for more than 15 years [24]. Six colonies roost in abandoned buildings surrounded by primary and secondary rain forest at “La Selva” Biological Station (10°25′N; 84°00′W, Province Heredia).

### Estimating age

As part of a long-term project all individuals are marked with numbered and coloured plastic rings (AC Hughes LTD., Middlesex, UK, size XCL) and the date of the first capture is reported in a database. Most juveniles were caught and marked during their year of birth in summer, so that we know the age of individuals that were marked as juveniles. However, new unmarked adult individuals are occasionally caught, of which we do not have direct information about the year of birth. The age of these individuals was therefore estimated with the help of the knowledge that we have on the social structure of this species: Following the approach outlined in Greiner and colleagues [24], we assumed that unmarked females found in colonies after the lactation period are immigrants that were born either in the year of capture if caught in winter (age = 1) or in the previous year (age = 2) if caught in summer. This is based on the finding that female juveniles disperse after getting weaned in late summer to avoid inbreeding with their father, while male offspring remain in their natal colony [25]. We assigned the age of males that were marked as adults in the same way, assuming that unmarked adult males were not caught during their juvenile stage, as they were probably roosting in a more remote place of the colony. Such assignments were done for in total 32 out of 128 individuals (17 aged as 1 year old; 10 aged 2 years; 1 aged 3 years; 1 aged 4 years; 2 aged 5; 1 aged 6 years).

### Estimating survival

Survival was estimated from census data collected during two seasons each year (once during sample collection in November/December, and once during July/August the next year) during the day. In the colony, individuals do not form clusters but remain at a minimum distance of 5–8 cm from each other [26]. This allowed us to identify bats either by taking photographs of the colony and identifying the individuals later on by matching the coloured rings with the database, or by identifying the colour of rings with short-range binoculars. Colony members were well habituated to the presence of an observer, enabling us to identify and observe individuals from a distance of 3–8 meters.

In order to estimate survival, we checked whether an individual that was sampled in November/December was reported to be present in the colony in July/August the next year. The probability for a marked male being present in the colony on a given day was previously determined as averaging 98% [27], while females once they immigrated in a colony had a probability of 91% to be present in the colony [28]. We visited each colony at least twice during each season with a break of at least one week between visits. If an individual was not encountered during both visits and in the subsequent season, we assumed that this individual disappeared permanently from the colony after the last observation, indicating that the individual has died. No individual re-appeared under these conditions and we therefore set disappearance equal to mortality. As sampling took place in November/December, after the dispersal event of subadult females, all sampled females were immigrants from previous summers. Therefore, we assume that disappearance of females from the colony in the time period after the sampling is caused by mortality and not by dispersal [25]. We could largely exclude human disturbance as a reason for individuals to disappear, as the access to colonies was restricted. However, at one roosting site, a former building, space was temporarily used as a storing room, which led to the disappearance of the whole colony in 2012. We therefore excluded datapoints from these individuals (N = 12) collected in 2012 from the analysis on the effect of immune parameters on survival, as disappearance could not be attributed to mortality in this situation. In order to rule out capturing and handling as a disturbance that might cause sampled bats to leave the colony, we occasionally checked for the presence of individuals the day after the sampling event. None of the sampled bats have been found to have disappeared from the colony 24 h after sample collection.

### Blood sample collection

Blood samples were collected in November and December 2010 and 2011. We captured bats when they returned to their roost at dusk between 0515 and 0615 hours using monofilament nylon mist nets (2.5 m height, 3 or 6 m length, Ecotone, Gdynia, Poland) placed at a minimum distance of 2 m from their daytime roosts. In one roost, bats were captured with hand nets. None of the females were pregnant or lactating, since the capturing took place outside the reproductive season. We identified the sex of all bats and weighed all individuals using an electronic balance (accuracy at 0.1 g) and forearm length was measured using a calliper. We calculated Body Condition Index (BCI) as body mass/forearm length, a standard method to measure energy reserves relative to a structural element of the body in bats [29], [30]. We took approximately 50 µl of blood from a total of 79 clinically healthy individuals (55 in 2010 and 24 in 2011) within 20 min after capture by puncturing the antebrachial vein with a sterile needle and collecting the blood with sterile heparinised capillaries. One drop was used for preparing blood smears on glass slides (Microscope Slides (76×26 mm), cut edges, Menzel, 38116 Braunschweig, Germany), while 10 µl blood was used to quantify the trypanosomes (see below). From 64 out of 79 individuals, we gained sufficiently large blood volumes to extract plasma after centrifugation at 11,500 rpm for 10 min. Plasma was stored at −80°C in two aliquots, one for BKA measurement, one for quantification of immunoglobulin G (IgG) concentration. Droppings excreted by bats during the sampling process were collected for the identification of helminth infections and stored in formalin until further analysis. After sampling, bats were immediately released at the site of capture.

### White blood cell counts

As in previous studies on vertebrate immune systems, we assumed that the number of white blood cells (WBC) represents a proxy of an animal’s investment into cellular immunity. We manually estimated total WBC counts by counting 10 visual fields with a microscope under 200x magnification on blood smears stained with May-Gruenwald’s (#T863.2, Carl Roth GmbH) and Giemsa (#T862.1, Carl Roth GmbH) solution, a method that has been used before in studies on mammalian immunology, including bats [31]–[34]. We obtained WBC counts for 47 individuals in 2010 and 23 individuals in 2011. In order to increase the sample size, we additionally analysed blood smears of individuals sampled from the same population in 2009 (N = 24; [35]) and 2012 (N = 24). As the rest of the blood sample from these years were used for other research purposes, no other immunological parameters than WBC counts were available from these additional individuals. We validated this method of estimating WBC counts by repeating counts on 40 smears, finding no difference between the first and second count (paired Wilcoxon signed-rank test; V = 358.5; p = 0.69) and the counts being correlated (Spearman’s rank correlation; rho = 0.64; p<0.001).

### Bacterial killing ability

We measured the constitutive innate immune function by assessing the bacterial killing ability of plasma (BKA) against *Escherichia coli* [36], an assay that has been used in bats before [34], [37]. Measurements were obtained for 40 individuals in 2010 and 24 individuals in 2011. Individual plasma samples were diluted 1∶20 with a CO_2_-independent media (#18045, Gibco-Invitrogen, CA) enriched with 4 mM L-Glutamine (#25030, Gibco-Invitrogen, CA) and 5% Fetal Calf Serum (#S0115, Biochrom AG). Ten µl of a suspension of living *Escherichia coli* (ATCC #8739) was added to 140 µl of diluted sample. Previously, the number of bacteria in the suspension was adjusted in order that 50 µl of plasma-bacteria mixtures produces approximately 200 colonies.

The plasma-bacteria mixtures were then incubated for 30 min at 37°C and afterwards, 50 µl aliquots were spread onto Tryptic Soy Agar plates (#CP70.1, Carl Roth GmbH) in duplicates. To obtain the number of bacteria that we had before the interaction with the plasma, we diluted the bacterial suspension with 140 µl media without plasma and plated the mixture in duplicates without previous incubation. We incubated the plates overnight at 37°C and counted the number of colonies formed the following day. The bacterial killing activity was calculated as the proportion of bacteria killed during the incubation, corresponding to 1 – (average of the viable bacteria after incubation/the initial number of bacteria), and the average was taken from two plates per sample [34]. BKAs did not differ within individuals as calculated from the duplicates (paired Wilcoxon signed-rank test; V = 446.5; p = 0.27) Duplicates were highly correlated (Spearman’s rank correlation; rho = 0.97; p<0.001).

### Immunoglobulin G concentration

Immunoglobulin G (IgG) levels were quantified for 39 individuals in 2010 and 23 individuals in 2011 using a Protein G ELISA [38]. Protein G, a streptococcal protein, binds IgG from a number of different wildlife species [39], and it has been validated to be used in routine serological testing of different bat species for specific antibody levels [40]. Following initial checkerboard titrations, we coated microtiter plates (Nunc-ImmunoTM Plate, NUNCTM Brand Products) with 100 µl of diluted plasma sample (diluted 1∶10.000 in 50 mM NaHCO_3_, pH 9,5) and incubated for 1 hour at 37°C. After incubation, plates were washed twice with Tris-buffered saline-Tween20 solution (TBS-Tween20). Two hundred µl of 1% gelatine solution (#104070, Merck) was added to each well for blocking non-specific reagent binding and plates were incubated for 30 minutes at 37°C. Subsequently, plates were washed as described above and 100 µl of Protein G– horseradish peroxidase conjugate (#P-21041, Invitrogen) at a dilution of 1∶12.000 in TBS-Tween20 solution was added to each well. Following 30-minute incubation at room temperature, the plates were washed five times in TBS-Tween20. Wells were then submerged with 100 µl of freshly prepared TMB solution [10% 3,3′,5,5′-tetramethylbenzidine (#0411-01, SouthernBiotech) dissolved in DMSO (#D5879, Sigma Aldrich) was diluted 1∶100 in phosphate-citric-buffer and mixed with 30% H_2_O_2_ (#7475456, Hedinger)] and the reaction was stopped with 1 M acid sulphuric after eight minutes. Plates were then immediately transferred to a microplate reader (Biotek) and the absorption was read at 450 nm. According to the Beer–Lambert law, antibody concentration is directly proportional to the absorption. We therefore conducted statistical analysis on the mean optical density (OD) of duplicates measured by the microplate reader for each sample.

### Estimating parasite infections

In order to quantify trypanosome infections of individual bats, we transferred 10 µl of blood into a micro-capillary and centrifuged it at 11′500 rpm for 3 minutes and 20 seconds (5,396 g) with a Compur M1100 microhaematocrit centrifuge (Bayer, 51368 Leverkusen, Germany; [35]). Trypanosomes were detected visually in 80 individuals after centrifugation of the blood by examining the buffy coat under the microscope at 200x magnification. A total of 35 out of 80 bats were infected with trypanosomes.

For the identification of helminth infections, we conducted faecal egg counts (FEC) using the McMaster flotation technique [41] with potassium iodide 1.5 g/ml solution on samples obtained from 47 individuals (7 in 2009, 28 in 2010 and 13 in 2011) [42]. We classified helminth eggs as cestodes, nematodes or trematodes according to size and morphology [43]. Individuals were then assigned to be either infected or not infected by trypanosomes and/or certain types of helminths. In droppings of 28 out of 47 bats, we encountered eggs of nematode, while only two bats showed signs of infection with trematodes and none signs of cestode infections. Therefore, we focused our statistical analysis on trypanosome and nematode infections to investigate the influence of parasites on immune parameters and survival.

### Statistical Analysis

In total, we acquired WBC counts of 118 individuals, BKA of 64 individuals and IgG concentrations of 62 individuals (Table S1). In 18 out of 118 individuals, we counted WBCs in two years and in 4 individuals WBC counts were obtained from three years. In 8 out of 64 individuals, we estimated BKAs in two consecutive years, while IgGs were measured twice in 7 out of 62 individuals. To test if there are longevity-related changes in immune parameters due to selective disappearance or individual immunosenescence, we used both a cross-sectional and longitudinal approach: We fitted a generalised linear model (GLM) on the cross-sectional dataset for each immune parameter, considering age (1–7 years), BCI (continuous) and sex (2 levels: males, females) as covariates. To fit the GLM on WBC count, we used a negative binomial distribution because Poisson distribution led to overdispersion. To fit the GLM on BKA and IgG concentration, we used a Gaussian distribution after applying a power transformation on the response variable to fulfil linear model assumptions [44].

To conduct the longitudinal analysis, we performed three tests. First, we asked whether individuals that were sampled twice in two consecutive years showed a general change in immune parameters using Wilcoxon signed rank tests. Of the 4 individuals which were sampled three times for WBC count, we used both changes from year 1 to year 2 and from year 2 to year 3 in the analysis. Second, we tested how departure from average immune condition influences survival by fitting logistic regression models. In these new GLMs, the dependent variable was the binary variable indicating the survival status (1 = survive, 0 = dead) and the covariates were the sex, age and the standardised residuals derived from GLM performed on the cross-sectional dataset for the immune parameter being considered. The standardised residuals of WBC count, BKA and IgGs represent the immune state of individuals once the effect of age, BCI and sex is accounted for. Third, we used Fisher’s exact tests to test if individuals that survived until the next season were more likely to be infected with trypanosomes or nematodes than individuals that did not survive, and we finally tested if infected individuals differed in immune parameters in comparison to uninfected bats using Mann-Whitney U tests.

All statistical analyses were performed with the free open-source statistical software R 3.0.2 [45]. We used the package “MASS” [46] for fitting negative binominal distributions and the package “car” [47] to apply power transformations on the data.

## Results

### Effect of age on immune parameters

**Figure 1:**
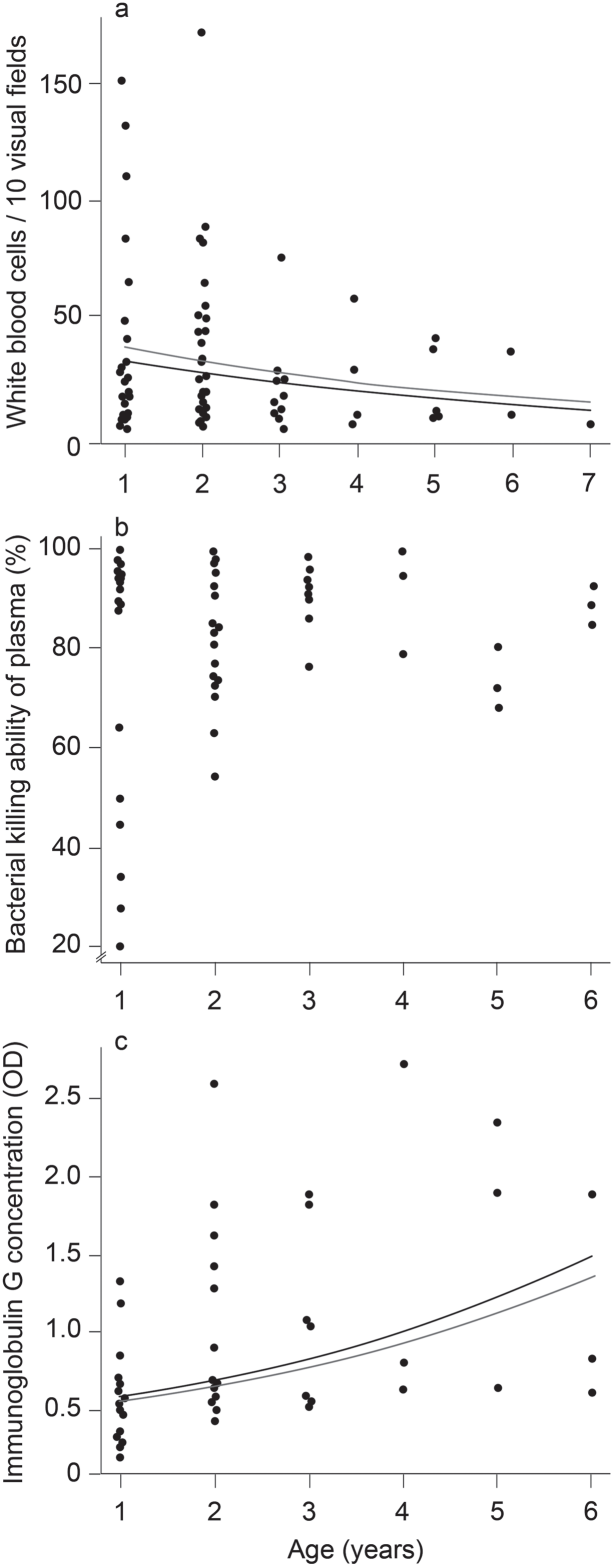
Cross-sectional association between age and immune parameters in *S. bilineata*. Dots indicate individual measures while solid lines outline the expected values predicted from the statistical models for males (grey line) and females (black line). White blood cell (WBC) count correlated negatively with age (a), while bacterial killing ability (BKA) of the plasma did not change with age (b). Concentration of immunoglobulins (IgG) correlated positively with age (c).

**Figure 2:**
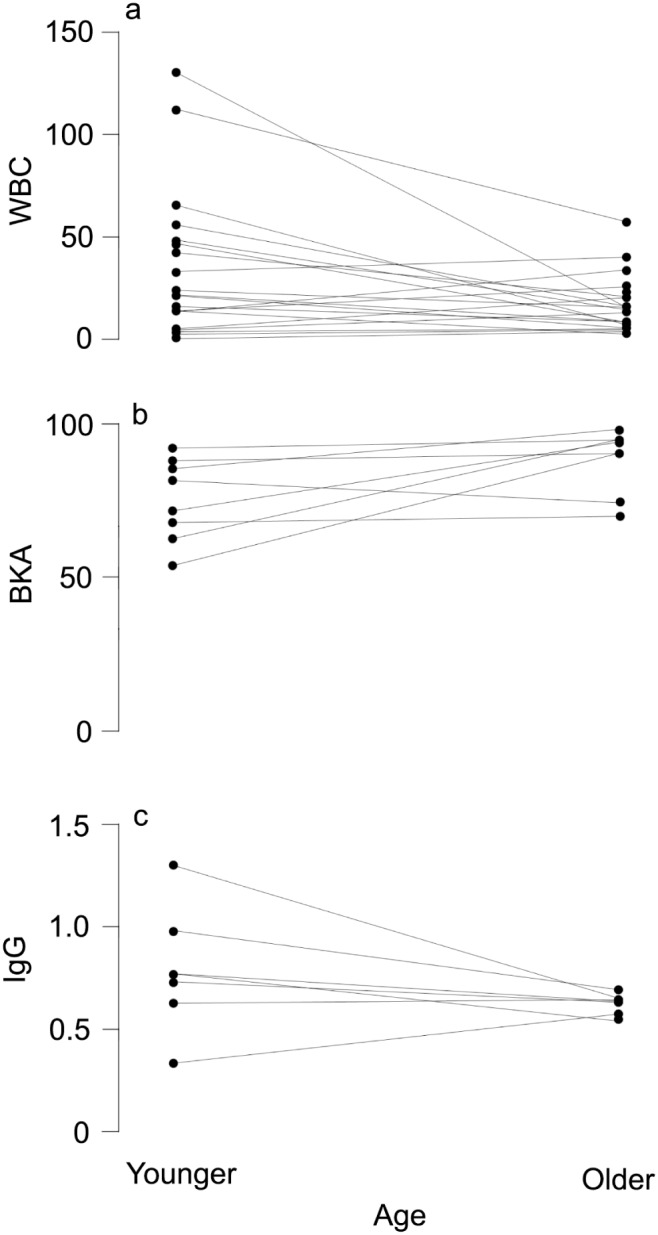
Changes in immune parameters within individual *S. bilineata*. Data of the same individual are connected via a solid line. White blood cell (WBC) count decreased with age (a) while there was no change in bacterial killing ability (BKA) of the plasma (b) and immunoglobulig G (IgG) concentration (c).

The analysis of our cross-sectional dataset shows age-related changes in immune parameters, i.e. we found that total WBC counts decreased with increasing age of individual *Saccopteryx bilineata* (GLM: X^2^_1_ = 4.26, p = 0.039; Fig. 1a). In contrast, IgGs increased with age (GLM: F_1,51_ = 11.2, p = 0.002; Fig. 1c), and BKAs were not correlated with age of individuals (GLM: F_1,52_ = 0.150, p = 0.70; Fig. 1b). The body condition of individuals was not related with any of the immune parameters (WBC: X^2^_1_ = 1.77, p = 0.18; BKA: F_1,52_ = 1.41, p = 0.24; IgGs: F_1,49_ = 0.244, p = 0.62). There was also no evidence for sex-specific difference in immunological parameters (WBC: X^2^_1_ = 0.74, p = 0.39; BKA: X^2^_1_ = 0.19, p = 0.67; IgGs: F_1,51_ = 0.185, p = 0.67). In our longitudinal data set, total WBC counts decreased with age within individuals (Wilcoxon signed-rank test: V = 201; N = 22; p = 0.016; Fig. 2a) and BKA tended to increase (V = 4; N = 8; p = 0.055; Fig. 2b). We did not observe any age-related changes of IgG concentration (V = 22; N = 7; p = 0.21; Fig. 2c).

### Immune parameters as predictors for survival

**Figure 3:**
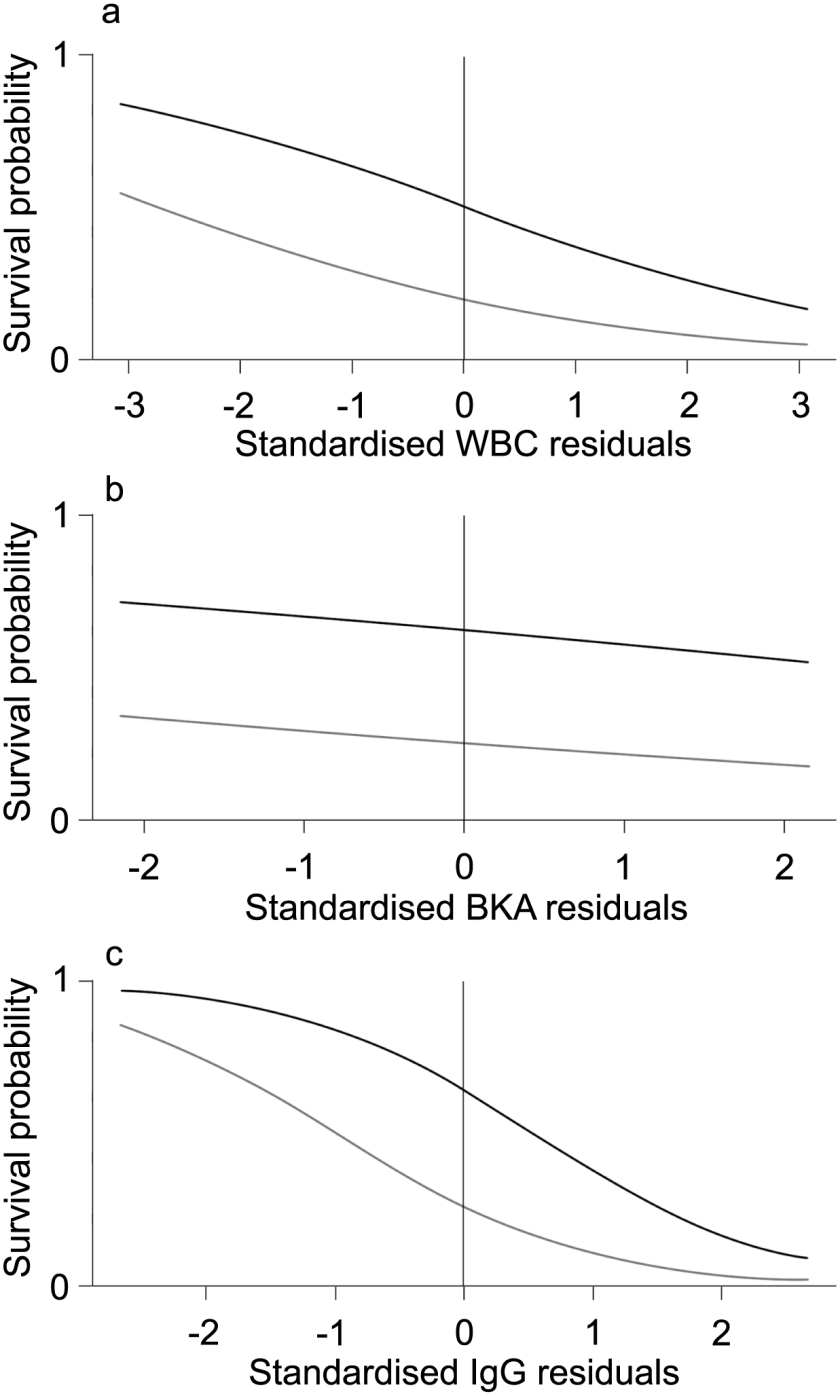
Survival probability of individuals with residual immune parameters. Standardised residuals in immune parameters depict individuals’ age-, body condition- and sex-specific deviation from average white blood cell (WBC) count, bacterial killing ability (BKA) of the plasma and immunoglobulin G (IgG) concentration. Residuals are plotted against the survival probability (males in grey, females in black). Individuals with higher WBC count (a) and higher IgG concentration (c) have lower probabilities to survive until the next season than individuals with low WBC count and low IgG concentration. BKA was not correlated with survival (b).

We found that mortality was high in the population, with 50% (43 out of 85) of all individuals older than one year not being observed six months after the last census. The probability of survival was related to immune parameters. Indeed, survival could be predicted by residuals of WBC (X^2^_1_ = 4.36, p = 0.037; Fig. 3a) and of IgGs (X^2^_1_ = 8.87, p = 0.003; Fig. 3c), but not by residuals of BKA (X^2^_1_ = 0.49, p = 0.48; Fig. 3b). Individuals with WBC counts and IgG concentrations that were one standard deviation above the average had 1.7 and 2.9 times less chance to survive to the next season than average individuals of the same age, BCI and sex. In general, it was 5 times more likely for females to survive until the next season than for males (with WBC: Odd-Ratio = 4.14, X^2^_1_ = 7.51, p = 0.006; with BKA: Odd-Ratio = 4.92, X^2^_1_ = 6.36; p = 0.012; with IgG: Odd-Ratio = 5.19, X^2^_1_ = 5.63; p = 0.018). There was no connection between survival probability and BCI (with WBC: X^2^_1_ = 0.46, p = 0.50; with BKA: X^2^_1_<0.001, p = 0.99; with IgG: X^2^_1_ = 0.01, p = 0.93). There was also no relationship between survival and age (with WBC: X^2^_1_ = 0.03, p = 0.87; with BKA: X^2^_1_ = 1.12, p = 0.29; with IgG: X^2^_1_ = 1.03, p = 0.31).

### Parasite infection, immune parameters and survival

We found no evidence that the observed mortality or immunosenescence was mediated by parasite infections. WBC counts did not differ between individuals infected and not infected by trypanosomes (Mann-Whitney U test: W = 443, N = 54, p = 0.66) or nematodes (W = 136, N = 30, p = 0.73). BKA was higher in bats with trypanosome infections than in uninfected individuals (W = 53, N = 24, p = 0.030) and tended to be higher in bats infected with nematodes too (W = 61, N = 26, p = 0.073). IgGs did not differ between uninfected bats and individuals infected with trypanosomes (W = 145, N = 26, p = 0.32) or nematodes (W = 96, N = 27, p = 0.75). Overall, survival probability did not differ between uninfected bats and individuals infected with trypanosomes (Fisher Exact Test: p = 0.23) or nematodes (p>0.99).

## Discussion

Understanding how immune parameters vary with age and how they are traded against other costly life history traits is important in order to better understand the evolution of the immune system in general and to predict the influence of environmental conditions on the health status of free-living individuals and populations. Here, we obtained data on several aspects of the immune system from free-ranging greater sac-winged bats (*Saccopteryx bilineata*). Focal individuals ranged in age between 1 and 7 years, which constitute the average lifespan of this species [24], [48]. To our knowledge, this is the first study that addresses the question how aspects of the immune system are connected with age and survival in a free-ranging mammal using longitudinal data. Specifically, we found evidence for i) senescence acting on some immune parameters and ii) a negative correlation between immune components and survival.

First, our study brings support for the idea that some immune components, as most other physiological processes, may decrease due to senescence of the organism (immunosenescence; [12]). Indeed, we found that white blood cell (WBC) counts decreased, while immunoglobulin G (IgG) concentration increased with increasing age in both males and females. One straight-forward explanation for this finding would be that individuals with leucocytosis (high WBC counts) disappear from the population, as this proxy is a widely used clinical marker both in human and in veterinarian medicine to diagnose on-going infections. We therefore tested for a correlation between immune parameters and infections with parasites. We found that endo- and hemoparasites were not generally associated with WBC counts or IgG concentration, although the constitutive humoral immune competence measured by bacterial killing ability (BKA) of the plasma was higher in individuals infected with trypanosomes and tended to be higher with nematode infections. Moreover, albeit our protocol only allowed for the identification of helminths and blood parasites, all animals we sampled did not show any clinical signs of disease, suggesting that high WBC counts and IgG concentrations were not likely to be caused by on-going infections from other, potentially more virulent pathogens such as viruses, bacteria, fungi or other parasites.

The decrease in WBC count with age is therefore more likely to be caused by immunosenescence. Indeed, we found decreasing WBC counts in our longitudinal datasets, which supports that this pattern most likely corresponds to the influence of age within individuals and is not an artefact caused by selective disappearance of older individuals with high WBC counts [21]. Several mechanisms of quantitative cellular immunosenescence have been found in studies on human gerontology. For example, thymic involution results in decline of the lymphoid precursor T- and B cells [49], while decrease in the function and number of hematopoietic stem cells also can affect the number of different circulating immune cells, both lymphoid and myeloid [50], [51]. Besides age associated decreases in the number of adaptive and innate immune cells, functional immunosenescence was also reported in humans [49], [52]. In contrast to leucocytes, we did not find support for immunosenescence acting upon the two other humoral immune parameters we measured – BKA and IgG concentration –, neither in our cross-sectional nor in our longitudinal data, results which are consistent with previous findings on humans [53], [54].

Second, our study also provides evidence for a trade-off between immunity and survival. Individuals with above average WBC counts and IgG concentrations were less likely to survive from one census to the next. Again, this could be a consequence of increased WBC and IgG levels mirroring an on-going infection or infestation. However, we found that parasite infections did not correlate with survival probability in *S. bilineata*, suggesting that the mere allocation of resources to the immune system might be costly in terms of survival. The existence of this trade-off should trigger a selective disappearance of individuals with high WBC or IgG with time and result in a decrease in those immune parameters with age in the population (between-individual effect), independently of the effect of immunosenescence (within-individual effect). This prediction is consistent with our observations of age-related decreases in WBC in the cross-sectional data set. Yet, older individuals had higher IgG concentrations than young ones, which supports the hypothesis that high antibody concentration may be a consequence rather than a cause of longevity [11]. Thus, higher IgG concentrations at an older age may reflect cumulative exposure to pathogens over time [54].

Various proximate mechanisms can account for the costs associated with high WBC counts. For example, oxidative stress, the imbalance between highly reactive pro-oxidants and neutralising antioxidants, might be one such proximate mechanism. Oxidative stress leads to the damage of cell components, to accelerated senescence of cells and eventually senescence and death of the organism [55]–[57]. WBCs produce pro-oxidants to directly kill pathogens [58] and to enhance the activation of T-lymphocytes [58], [59]. Therefore, high WBC count may be associated with increased oxidative stress and oxidative damage. In a previous experiment, we showed that a cellular immune response causes an increase in concentration of damaging pro-oxidants in blood of the Neotropical short-tailed fruit bat, *Carollia perspicillata*, and that the number of immune cells is positively correlated with measures of oxidative stress [60]. Given that cells become less resistant to oxidative stress with increasing age [61], older individuals may be more affected by oxidative stress induced by WBCs. Overall high levels of WBCs may therefore be evolutionary selected against to minimise oxidative damage and early senescence in animals.

A second proximate mechanisms underpinning the cost of maintaining high levels of immune functions is that it may increase the risk of autoimmunity [62], [63]. Autoimmunity is mainly promoted by the increased production of antinuclear antibodies that usually bind foreign proteins, but may also be produced against antigens of the organism itself. In contrast to Graham and colleagues [11] we found that individuals with high IgG concentrations were less likely to survive the following six months than individuals with low concentrations, which is consistent with the idea of increased risk of autoimmunity. IgG concentrations being higher in older bats, but not increasing with age within individual, furthermore suggests selective disappearance of individuals with low IgG concentrations from the population.

In general, immune effectors which have either high developmental, maintenance or pathological costs (e.g. immune cells, immunoglobulins or acute phase proteins; [23]), seem to be traded against longevity. In contrast, we observed that relatively low-cost constitutive innate immunity - measured here as the bacterial killing ability (BKA) of the plasma against *Escherichia coli* - were not connected with age, neither in the cross-sectional nor longitudinal data set. Also, BKA was not associated with survival probability.

The relationship between immunity and longevity may however be different in populations or species under higher pathogen and parasite pressure than *S. bilineata*. On the one hand, high levels of infection could hide the trade-off between immunity and survival in situations for which high levels of WBC and IgG are cues of ongoing, acute or chronic infection that can precede the death of individuals. On the other hand, in a highly pathogenic environment the trade-off may express itself differently because the cost of investing resources in the immune system may be overcome by benefits of surviving those infections. This could explain for instance, why immunoglobulin concentration was positively associated with survival in Soay sheep [11] and recapture rate in house martins (*Delichon urbica*) [64] but negatively associated with survival in *S. bilineata* (this study). Indeed, Soay sheep infected by nematodes may face high mortality risks during harsh winters [65], which may even lead to population crashes [66]. Furthermore, longitudinal studies on the same population found age-related changes in parasite resistance [67], [68]. These findings potentially indicate that endoparasite infections may involve higher costs in Soay sheep than in *S. bilineata*, especially when resources are limited.

Besides immune components being connected with survival, we also found that females have a five times higher chance to survive during the next six months than males. Our results are consistent with a recent study showing male senescence in fitness and reproduction in *S. bilineata* [24]. In most mammals, females have higher survival chances than males, which in monomorphic species is frequently hypothesized to be caused by risky behaviour (e.g. [69]) or increased investment in reproduction in males. Indeed, mass-specific field metabolic rate of males increases with harem size in *S. bilineata* [70], suggesting an overall high cost of courtship display in this species, which may explain why males have lower chances of survival than females.

Variations in immune components within bat species may be important in explaining the susceptibility of individuals and populations to pathogens and also their reservoir-competence. For example, it has been found that bats that are immune suppressed are more likely to develop the white-nose syndrome caused by the fungus *Pseudogymnoascus destructans* in North America [71], [72]. In this context, our study suggests that older individuals with fewer WBCs may be more susceptible to this lethal disease. If older individuals are indeed more prone to get infected by virulent pathogens, then understanding how human-driven environmental changes impact the age structure of wild populations may help to better understand the dynamic of harmful zoonotic diseases. More generally, our findings confirm that longitudinal studies on free-ranging populations should be encouraged in order to tackle both fundamental and applied questions related to the senescence of organisms.

## Supporting Information

Table S1 **Individual data on age, sex, body mass, forearm length, white blood cell (WBC) count, bacterial killing ability of the plasma (BKA) and immunoglobulin G (IgG) concentration of 118 individual greater sac-winged bats (** ***Saccopteryx bilineata*** **).**
(XLS)
